# Coconut Lethal Yellowing Diseases: A Phytoplasma Threat to Palms of Global Economic and Social Significance

**DOI:** 10.3389/fpls.2016.01521

**Published:** 2016-10-26

**Authors:** Geoff M. Gurr, Anne C. Johnson, Gavin J. Ash, Bree A. L. Wilson, Mark M. Ero, Carmel A. Pilotti, Charles F. Dewhurst, Minsheng S. You

**Affiliations:** ^1^State Key Laboratory of Ecological Pest Control for Fujian and Taiwan Crops, Fujain Agriculture and Forestry UniversityFuzhou, China; ^2^Institute of Applied Ecology, College of Plant Protection, Fujian Agriculture and Forestry UniversityFuzhou, China; ^3^Graham Centre of Agricultural Innovation, Charles Sturt UniversityOrange, NSW, Australia; ^4^Research and Innovation Division, Centre for Crop Health, Institute for Agriculture and the Environment, University of Southern QueenslandToowoomba, QLD, Australia; ^5^PNG Oil Palm Research AssociationKimbe, Papua New Guinea; ^6^Formerly affiliated with the PNG Oil Palm Research AssociationKimbe, Papua New Guinea

**Keywords:** phytoplasma, insect vector, plant pathology, phytosanitation, quarantine, host plant resistance, CRISPR, LAMP

## Abstract

The recent discovery of Bogia coconut syndrome in Papua New Guinea (PNG) is the first report of a lethal yellowing disease (LYD) in Oceania. Numerous outbreaks of LYDs of coconut have been recorded in the Caribbean and Africa since the late Nineteenth century and have caused the death of millions of palms across several continents during the Twentieth century. Despite the severity of economic losses, it was only in the 1970s that the causes of LYDs were identified as phytoplasmas, a group of insect-transmitted bacteria associated with diseases in many other economically important crop species. Since the development of polymerase chain reaction (PCR) technology, knowledge of LYDs epidemiology, ecology and vectors has grown rapidly. There is no economically viable treatment for LYDs and vector-based management is hampered by the fact that vectors have been positively identified in very few cases despite many attempted transmission trials. Some varieties and hybrids of coconut palm are known to be less susceptible to LYD but none are completely resistant. Optimal and current management of LYD is through strict quarantine, prompt detection and destruction of symptomatic palms, and replanting with less susceptible varieties or crop species. Advances in technology such as loop mediated isothermal amplification (LAMP) for detection and tracking of phytoplasma DNA in plants and insects, remote sensing for identifying symptomatic palms, and the advent of clustered regularly interspaced short palindromic repeats (CRISPR)-based tools for gene editing and plant breeding are likely to allow rapid progress in taxonomy as well as understanding and managing LYD phytoplasma pathosystems.

## Introduction

**Figure 1:**
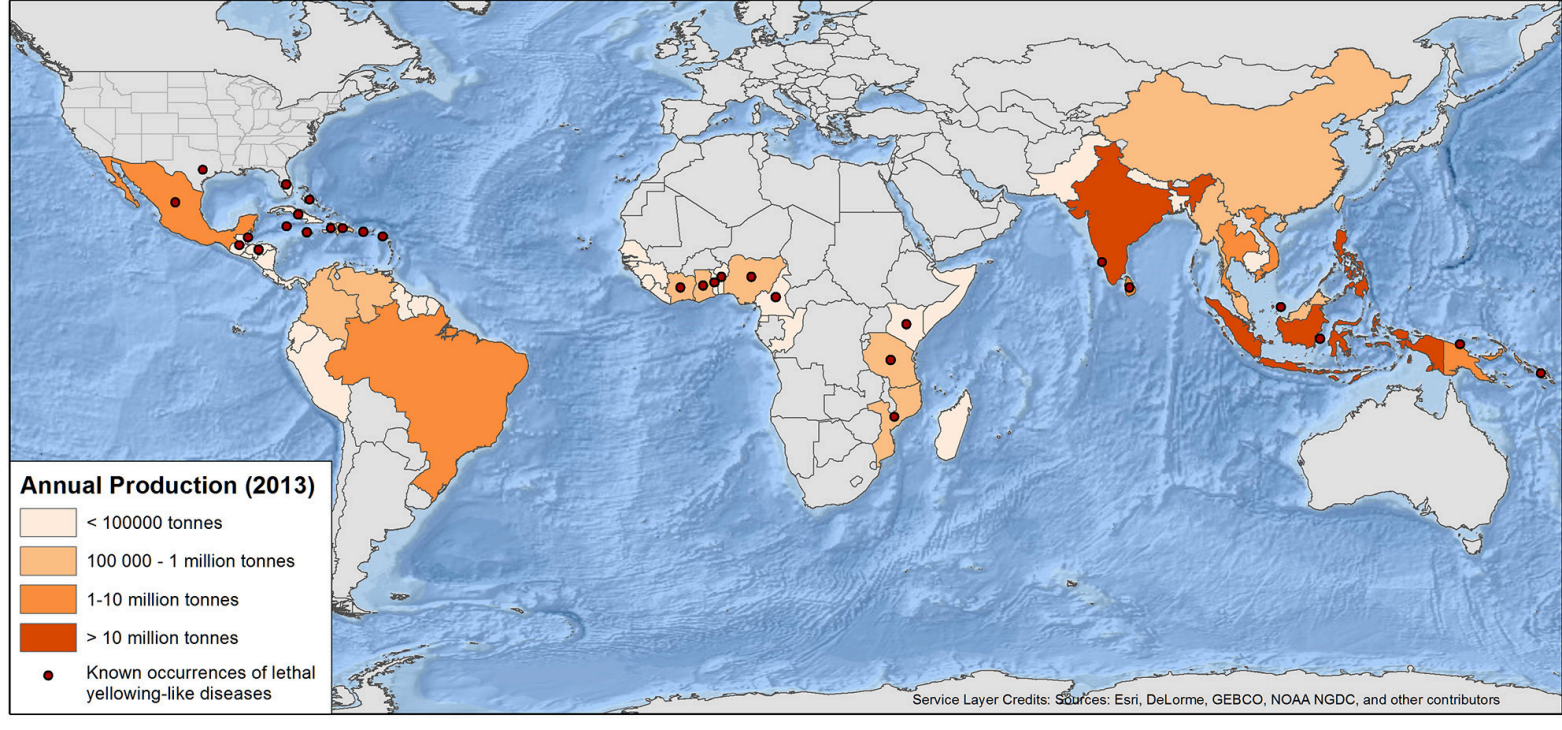
**World map of annual coconut production and current published occurrences of lethal yellowing-type diseases of palms (see Table 1 and text for full detail and caveats) production data from FAO (2016)**.

Phytoplasma-associated diseases occur in many plant species and, reflecting their great economic importance, there is a large body of literature available focusing on phytoplasmas in agriculture (Bertaccini et al., 2014), vectors (Weintraub and Beanland, 2006; Weintraub, 2007), biology (Christensen et al., 2005; Namba, 2011; Maejima et al., 2014; Bertaccini, 2015), diagnosis and classification (Lee et al., 1998b, 2000; Bertaccini, 2007; Firrao et al., 2007; Duduk and Bertaccini, 2011; Harrison et al., 2014) and genomics (Kube, 2011). Among the more serious phytoplasma diseases are the lethal yellowing-like diseases (LYDs) of palms that have caused major outbreaks leading to the losses of millions of coconut and other palm species (Jones, 2002; Eziashi and Omamor, 2010). Lethal yellowing-like diseases, also called Lethal Yellowing Type Syndromes, Lethal Declines or Coconut Lethal Yellowing comprise of a complex of phytoplasma-associated coconut diseases found around the world (Figure 1, Table 1) that result in yellowing, wilting and death of palms. Reviews of LYDs include Danyo (2011), Dollet et al. (2009), Elliott (2009), Elliott and Harrison (2007), Eziashi and Omamor (2010), Harrison et al. (2014), Jones (2002), Ntushelo et al. (2013c), Oropeza et al. (2011), Ramjegathesh et al. (2012), Tsai and Harrison (2003), and Wilson (1987).

**Table 1:** **Current distribution and range of lethal yellowing-type diseases of palms^**a**^**.

**Location**	**Name**	**16Sr Group**	**Host**	**References**
**AMERICAS**				
Florida, USA Caribbean Basin (Antigua, Bahamas, Belize, Cayman Islands, Cuba, Dominican Republic, Guatemala, Haiti, Honduras, Jamaica, Mexico, St. Kitts & Nevis)	Coconut lethal yellowing (CLY) or lethal yellowing (LY) *Candidatus* Phytoplasma palmae (*Ca* P. palmae)	IV-A	Coconut palm (*Cocos nucifera*), and 38 other palm species (Table 2).	Harrison et al., 1999, 2002a, 2008; Ntushelo et al., 2013c
Mexico, Honduras	Yucatan coconut lethal decline, lethal yellowing disease	IV-B	*C. nucifera, Acrocomia aculeata* (Jacq.)	Ashburner et al., 1996; Tymon et al., 1998; Cordova et al., 2003; Roca et al., 2006; Ntushelo et al., 2013c
Mexico, Texas and Florida, USA, Puerto Rico	Texas Phoenix palm decline (TPPD) *C. palmata* yellows (CPY) phytoplasma or Sabal mexicana lethal decline	IV-D	*Phoenix canariensis, P. dactylifera, P. reclinata, P. roebelenii, P. sylvestria, Sabal palmetto, Syagrus romanzoffiana, Carludovica palmata, Sabal mexicana, Pseudophoenix sargentii, Pritchardia pacifica, Thrinaz radiata, Carpentaria acuminata, Caryota mitis, Roystonea* sp.	Harrison et al., 2002b, 2009; Vázquez-Euán et al., 2011; Ntushelo et al., 2013c; Córdova et al., 2014
Dominican Republic	CLY	IV-E^c^	*C. nucifera*	Martinez et al., 2008; Ntushelo et al., 2013c; Córdova et al., 2014
Florida	CLY	IV-F	*Washingtonia robusta, Phoenix dactylifera*^d^	Harrison et al., 2008; Ntushelo et al., 2013c
**AFRICA**				
Tanzania, Kenya	Coconut lethal disease (CLD)	IV-C^e^	*P. dactylifera C. nucifera*	Tymon et al., 1998; Córdova et al., 2014
Mozambique	CLD	IV-B IV-C XXII-A	*C. nucifera*	Córdova et al., 2014; Harrison et al., 2014; Bila et al., 2015a
^b^Nigeria	Awka disease	XXII-A	*C. nucifera*	Ekpo and Ojomo, 1990; Tymon et al., 1998; Wei et al., 2007; Osagie et al., 2015
^b^Ghana, Côte d'Ivoire, Nigeria, Togo, Cameroon, Benin	Cape St. Paul wilt, CSPW Keta disease, Kaincopé, Kribi disease or Côte d'Ivoire lethal yellowing disease	XXII-B	*C. nucifera*	Dabek et al., 1976; Tymon et al., 1998; Makarova et al., 2012; Harrison et al., 2014; Osagie et al., 2015; Arocha-Rosete et al., 2015
**ASIA**				
India	Kerala wilt disease	IV-C (disputed)	*C. nucifera*	Edwin and Mohankumar, 2007a Disputed by Manimekalai et al. (2010) and Mehdi et al. (2012)
India	Root (wilt) disease	XI-A XI-B XIV	*C. nucifera*	Manimekalai et al., 2014c; Yadav et al., 2015
Sri Lanka	Weligama coconut leaf wilt disease (WCLWD) *Ca*. P.oryzae	XI	*C. nucifera*	Perera et al., 2012; Kumara et al., 2015
Malaysia	Coconut yellow decline (CYD) *Ca*. P. cynodontis	XIV	*C. nucifera*	Nejat et al., 2009a,b
Malaysia	*Ca*. P. malaysianum	XXXII-B XXXII-C	*C. nucifera E. guineensis*	Nejat et al., 2013
Indonesia	“Kalimantan wilt” and “Natuna wilt” *Ca*. P. oryzae	XI XIII	*C. nucifera*	Harries, 2005; Warokka et al., 2006 cited by Mehdi et al. (2012)
**OCEANIA**				
Papua New Guinea	Bogia coconut syndrome (BCS) Banana wilt associated phytoplasma (BWAP)	IV	*C. nucifera* Banana (*Musa* sp.)	Kelly et al., 2011; Pilotti et al., 2014; Davis et al., 2015
Papua New Guinea, Solomon Islands	Banana wilt associated phytoplasma (BWAP)	XXII-A	*C. nucifera* Bananas (*Musa* sp.)^f^	Davis et al., 2015

*Similar tables can be found in earlier references including Bertaccini et al. (2014) and Mehdi et al. (2012). The classifications frequently change as new groups and subgroups are found and classified (Harrison et al., 2014)*.

a *Excludes 16Sr Group I and XI phytoplasmas which are associated with diseases affecting date palms (P. dactylifera) (Mehdi et al., 2012; Gurr et al., 2015)*.

b *Until the end of the 1990s, phytoplasmas associated with “maladie de Kaincopé” in Togo, “Awka wilt” in Nigeria and CSPWD in Ghana were thought to fall within the 16SrIV group (Tymon et al., 1998). Recently, they were included in a new group, 16SrXXII (Wei et al., 2007), incidentally cited as “Ca. P. cocosnigeriae,” where particularly the phytoplasma associated with “Awka wilt” was classified as a new subgroup designated to 16SrXXII-A (Tymon et al., 1998; IRPCM 2004). From Arocha-Rosete et al. (2014)*.

c *Subgroup 16SrIV-E is closely related to 16SrIV-B*.

d *P. dactylifera was dual infected with 16SrIV-A (Ntushelo et al., 2013c)*.

e Subgroup 16SrIV-C is quite distinct from other IV subgroups and is more closely related to western African groups (Ntushelo et al., 2013c)

f *Banana (a non-palm) included because a host shift by the phytoplasma is suspected from banana to coconut (Davis et al., 2015)*.

Importantly, however, all reviews published since 2010 have a narrow focus on particular geographical areas and diseases such as: Ghana and Mozambique (Danyo, 2011; Harrison et al., 2014); the Carribean (Ntushelo et al., 2013c); Southern Asia (Ramjegathesh et al., 2012); or the distribution of phytoplasmas within palms (Oropeza et al., 2011). Accordingly, this article provides an up-to-date and comprehensive synthesis of LYDs from a global perspective, covering methods for detection in plants and determining vector status of insects, management methods, as well as the fundamental biology of relevant phytoplasmas. Recent developments in phytoplasma research include, improved rapid detection technology, genome sequencing and knowledge of the biological relationships between the pathogen, vector and host plant which are informing effective management. We canvass the wider phytoplasma literature where this has relevance to LYD. The review is timely because a LYD—Bogia coconut syndrome (BCS) in Papua New Guinea (PNG)—has recently been reported from Oceania for the first time (Kelly et al., 2011), and LYDs remain a serious issue in other regions with, for example, a current outbreak in Mozambique threatening the livelihood of over three million people (Bila et al., 2015b). Since the advent of molecular approaches to study phytoplasmas, there have been frequent changes in taxonomy therefore this review also provides an update on the taxonomic status of the phytoplasma taxa associated with different LYDs.

## Lethal yellows disease pathosystems

### Coconut and the economic implications of LYD

Coconut palm (*Cocos nucifera* L.), is grown in 90 countries around the world, mostly in tropical areas, by an estimated 11 million farmers across 12 million hectares, with over 80% of production in Asia (Adkins et al., 2006; FAO, 2014). Coconut provides a staple food and serves as a cash crop in many developing countries; copra (coconut “meat”) being one of the few sources of cash income for many households (Bourke and Harwood, 2009). Without coconut, habitation on some atolls and islands would be unsustainable, as not only is coconut a major source of food but coconut vegetation is used in building houses and the coconut shells are used for common household use such as bowls and fuel (Ovasuru, 1994). A range of value-added products is made from coconut so improvements in coconut production have resulted in social and economic benefits for some of the world's poorest areas.

Accordingly, any outbreak of disease, particularly LYD results in environmental and economic upheaval. For example, an outbreak in Côte d'Ivoire destroyed over 350 ha of plantations with a loss of 12,000 t of copra/year, with a further 7000 ha under threat (Arocha-Rosete et al., 2014). Coconut palms do not yield nuts until many years after replanting so producers may be forced to either relocate or replant with other crops if palms become infected with LYD. In many developing countries, such as PNG, the ability to produce a cash crop for domestic and overseas export markets is closely linked with relieving rural poverty and aiding development. Accordingly, large scale crop loss and the resulting lack of income will result in more rural agriculturalists moving to urban areas so exacerbating rural poverty (Allen et al., 2005).

### Lethal yellowing diseases

Lethal yellowing diseases are often referred to as syndromes due to gross similarities in common symptoms though the sequence and detail of symptom progression can vary based on the phytoplasma group, geographical location, host species, and variety (Dollet et al., 2009; Harrison et al., 2014). Detailed symptoms of LYD are described in Arellano and Oropeza (1995), Bertaccini et al. (2014), Córdova et al. (2014), Dollet et al. (2009), CABI (2012), and McCoy et al. (1983). Typical symptoms of most LYDs are the shedding of both ripe and undeveloped fruit (Dollet et al., 2009; Bertaccini et al., 2014) whilst yellowing of fronds first shows at the tips of the fronds and moves back toward the main stem, starting with the oldest fronds and moving to the youngest, the fronds eventually falling from the stem (Dollet et al., 2009; Bertaccini et al., 2014).

Internationally, LYD is most widely reported from coconut (Figure 1). Caution is required, however, in assuming that coconut is the sole host in any region because this could simply reflect a lack of research in some locations. LYD-associated phytoplasmas are known to affect more than 30 other palm species (Table 2), including date palm (*Phoenix dactylifera* L.) and oil palm (*Elaeis guineensis* Jacq.) (Bertaccini et al., 2014). It is coconut, however, that the most devastating losses have occurred. In Jamaica, LYD killed 4.5 million of the 5.2 million coconut palms between 1961 and 1983 (Jones, 2002). In Africa, “Akwa wilt” in Nigeria, “Cape St. Paul Wilt” in Ghana and “Kain-cope disease” in Togo killed millions of coconut palms (Eziashi and Omamor, 2010). In Tanzania, an estimated 8 million coconut palms or 38% have been killed by “Lethal Disease” since the 1960s (Mugini, 2002). Paradoxically, “lethal” yellowing-type diseases are not always lethal (Ntushelo et al., 2013c), but can be equally economically devastating due to loss of yield, such as in India, where Root (wilt) disease (RWD) is a “non-lethal, debilitating malady” (Manimekalai et al., 2014b).

**Table 2:** **List of palm species in Florida known to be susceptible to LYD (16SrIV-A) from Harrison et al. (1999) and Howard (2001b)**.

**Scientific name**	**Common name**	**Origin**
*Adonidia merrillii*	Christmas palm	Philippines and region
*Aiphanes lindeniana*	–	Central and South America and Caribbean
*Allagoptera arenaria*	Seashore palm	South America
*Arenga engleri*	Dwarf sugar palm	Southeast Asia
*Borassus flabellifer*	Palmyra palm	India
*Caryota mitis*	Clustering fishtail palm	Southeast Asia
*Caryota rumphiana*	Giant fishtail palm	Southeast Asia
*Chelyocarpus chuco*	Round leaf palm	Brazil & Bolivia
*Cocos nucifera*	Coconut palm	Western Pacific
*Corypha elata*	Buri palm	India
*Crysophila warsecewiczii*	Rootspine palm	Central America
*Cyphophoenix nucele*	Lifou Palm	New Caledonia
*Dictyosperma album*	Princess palm	Madagascar
*Dypsis cabadae*	Cabada palm	Madagascar
*Dypsis decaryi*	Triangle palm	Madagascar
*Gaussia attenuata*	Puerto Rican Gaussia palm	Caribbean
*Howea belmoreana*	Belmore sentry palm	Western Pacific
*Howea forsteriana*	Kentia or Sentry palm	Western Pacific
*Hyophorbe verschafeltii*	Spindle palm	Madagascar
*Latania lontaroides*	Latan palm	Madagascar
*Livistona chinensis*	Chinese fan palm	China
*Livistona rotundifolia*	Footstool palm	Southeast Asia
*Nannorrhops ritchiana*	Mazari palm	Asia minor
*Phoenix canariensis*	Canary Island date palm	Canary Islands
*Phoenix dactylifera*	Edible date palm	North Africa
*Phoenix reclinata*	Senegal date palm	Africa
*Phoenix rupicola*	Cliff date palm	India
*Phoenix sylvestris*	Silver date palm	India
*Pritchardia affinis*	Kona palm	Hawaii
*Pritchardia pacifica*	Fiji island fan palm	Western Pacific
*Prichardia remota*	–	Hawaiian Islands
*Pritchardia thurstonii*	Thurston palm	Western Pacific
*Ravenea hildebrantii*	Dwarf Majesty Palm	Comoros
*Syafrus schizophylla*	Arikury palm	South America
*Trachycarpus fortunei*	Windmill palm	China
*Veitchia arecina*	Majesty Palm	Fiji, Vanuatu, Tonga
*Veitchia merillii*	Christmas palm	Western Pacific
*Veitchia mcdanielsi*	Sunshine palm	Western Pacific
*Veitchia montgomeryana*	Montgomery's palm	Western Pacific

### Origin and distribution of lethal yellowing diseases

Lethal yellowing was first observed in the Caribbean in the late 1800s (Johnson, 1912; Plavsic-Banjac et al., 1972). About the same time, reports of a similar disease came from Tanzania then Togo and Ghana (Ekpo and Ojomo, 1990; Dollet et al., 2009). In the Caribbean, by 1912, “Coconut bud rot” stopped the commercial production of coconuts in some areas (Johnson, 1912). Outbreaks of LYD have since occurred in Florida, Jamaica, southern Mexico, Honduras (Ashburner et al., 1996), Haiti, Cuba, Belize and the Bahamas (McCoy et al., 1983; CABI, 2012). Due to the similarity of LYDs in different parts of the world, a common cause was initially suspected, although there were doubts due to differences in symptom progression and varietal susceptibility. As identification and classification technology developed (Harrison et al., 1994; Tymon et al., 1997) various types of LYD were found to be associated with different groups and subgroups of phytoplasma (Figure 1, Table 1) (Harrison et al., 2008, 2014; Dollet et al., 2009; Eziashi and Omamor, 2010; Bertaccini et al., 2014).

Today LYDs continue to be serious in the Caribbean and Central America (EPPO/CABI, 1997; Myrie et al., 2006, 2012; CARDI, 2013; Ntushelo et al., 2013c). Lethal yellowing-like diseases are known to be present in Benin, Cameroon, Ghana, Kenya, Mozambique, Nigeria, Tanzania, and Togo (EPPO/CABI, 1997; Eziashi and Omamor, 2010), India (Edwin and Mohankumar, 2007a; Ramjegathesh et al., 2012), Sri Lanka (Perera et al., 2012), Indonesia (Dollet et al., 2009), and recently PNG (Kelly et al., 2011). The impact of LYD is particularly felt in communities of smallholder and subsistence farmers that rely nutritionally and economically on coconuts (Myrie et al., 2011). Compounding the impact of this group of diseases, the coconut industry is often poorly resourced for carrying out phytoplasma research. In many parts of the world where LYDs occur, research is piecemeal usually due to the intermittent funding inputs through foreign aid (Baudouin et al., 2009; Philippe et al., 2009).

The history of reported outbreaks suggests that LYD originated in the Caribbean (Johnson, 1912; Parthasarathy, 1974). In contrast, Ogle and Harries (2005) point out that for several hundred years after their introduction in the Sixteenth century, coconut palms in the Caribbean were healthy. Records of cattle imports from India into the Caribbean correspond temporally and geographically with early records of LYD occurrence in the Nineteenth century, which led to speculation that cattle fodder carried on ships may have harbored a vector carrying the phytoplasma. Similar records of cattle transportation from India into West African nations corresponding to LYD occurrence in Africa support the theory that LYD originated in Asia (Ogle and Harries, 2005) (Table 1). The more recent discovery of increasing numbers of phytoplasma groups, however, suggests diverse origins. For example, Vázquez-Euán et al. (2011) used observations of low mortality of Texas palmetto (*Sabal mexicana*) to suggest the phytoplasma subgroup (16SrIV-D) may have a native (Mexico) origin. In the Caribbean, the spread of a vector to new areas has resulted in LYD outbreaks suggesting a non-native origin of the disease (Brown et al., 2006; Elliott, 2009), whilst in PNG the putative vectors of the recent outbreak of BCS are all native species (Pilotti et al., 2014).

### Pathogen discovery and taxonomy

Early investigations into the cause of LYD searched primarily for viruses but also extended to fungi, bacteria, nematodes and even abiotic factors (Johnson, 1912; Arellano and Oropeza, 1995). Eventually electron microscopic examination of palms showing the earliest symptoms of LYD found mycoplasma-like bodies in diseased palms but not in healthy palms and the absence of any other potential causative agents (Beakbane et al., 1972; Plavsic-Banjac et al., 1972; Parthasarathy, 1974). The successful use of antibiotics to protect palms from infection confirmed that LYD symptoms were caused by a bacterium that at the time was referred to as mycoplasma-like-organisms (MLO). For many years after their discovery very little was known about the biology of MLOs and knowledge was often speculative (Plavsic-Banjac et al., 1972; Parthasarathy, 1974; Thomas, 1974). The term MLO was replaced by phytoplasma (International Committee on Systematic Bacteriology Subcommittee on the Taxonomy of Mollicutes, 1993, 1997) and later given the interim taxonomic status of “*Candidatus* Phytoplasma” with LYD associated phytoplasmas as *Ca*. P. palmae and *Ca*. P. cocostanzaniae (IRPCM, 2004). More recently *Ca*. P. palmicola has been added to the list (Harrison et al., 2014). Further history on the discovery and naming of phytoplasmas can be found in Duduk and Bertaccini (2011), Eden-Green (1995), Howard (2001b), and Lee et al. (2007).

Initially, LYDs were named after their symptoms and host (Howard, 2001b; Ntushelo et al., 2013b). Lethal yellowing-like diseases cannot be properly identified by their symptoms alone because other diseases may show similar symptoms to LYD, including mineral deficiencies, “hartrot” caused by trypanosomatids or “red ring” caused by nematodes (Dollet, 2002). Furthermore, there are also large variations in LYD symptomatology, thus positive identification of phytoplasmas is usually by polymerase chain reaction (PCR) (Lee et al., 2007; Dollet et al., 2009; Ntushelo et al., 2013b; Bertaccini et al., 2014; Harrison et al., 2014).

Phylogenetic analyses are used as the basis for taxonomy of phytoplasmas. Lee et al. (2000) provide a thorough review of the taxonomic system of phytoplasmas using universal oligonucleotides based on the conserved 16S rRNA gene sequences for PCR assays. Taxonomic classification is achieved by comparing the 16S ribosomal gene sequences where they are classified into groups and subgroups (Table 1) (Lee et al., 1998b, 2007). Bertaccini et al. (2014) listed 33 16Sr Groups and over 100 subgroups with more reported annually. A phytoplasma is classified as a new species when its 16S rRNA gene sequence has a < 97.5% similarity with another “Ca. Phytoplasma” species. However, despite this genetic similarity phytoplasmas may exhibit varied biological or ecological differences. All types of phytoplasmas have a range of subgroups that may cause mild or severe symptoms. Multiple infections can occur and the ratio of the infection can determine the severity of the symptoms (Seemuller et al., 2011). Further distinctions could be made through observations of antibody specificity, host range and vector transmission specificity and using the sequences of less conserved genes such as rp, secA, secY, tuf, groEL, and the 16S-23S rRNA spacer regions. These discriminating tools can be used for characterization and epidemiological studies and even identification for quarantine purposes (Hodgetts et al., 2008, 2009; Duduk and Bertaccini, 2011; Mitrović et al., 2011; Fránová et al., 2013; Ntushelo et al., 2013a).

Phytoplasmas associated with lethal yellowing-type diseases of palms are most commonly placed in the 16SrIV group although some have now been reclassified into group 16SrXXII (Lee et al., 1998b; Harrison et al., 2014), 16SrI, 16SrXI, and 16SrXIV (Bertaccini et al., 2014). The 16SrIV group is sub-divided into A-F reflecting the genetic variation and the variety of plant hosts and vectors (Harrison et al., 2002a,b, 2008; Brown et al., 2006; Martinez et al., 2008; Vázquez-Euán et al., 2011). The diversity in the characteristics of phytoplasma subgroups that are associated with LYDs include host range, symptoms and vector(s). For example, whilst LY in Florida (16SrIV-A) mainly affected palms 18 months and older, other outbreaks of LYD (subgroup not given) affected bearing and non-bearing palms (Carter, 1966; Harrison et al., 2002a). BCS in PNG is similar to other LYD, yet there are differences in the symptoms, such as the absence of inflorescence necrosis, and both old and young palms are affected (Kelly et al., 2011).

It is common to show diversity in phytoplasmas that overlap geographically. A study in Mozambique showed the existence of three different groups in one area: one related to the West African subgroup (XXII-B), another to the East African subgroup (IV-C) and a novel unclassified coconut-associated phytoplasma (Bila et al., 2015a). Similarly in Malaysia, LYD symptoms were associated with phytoplasmas classified as being from the 16SrXIV and 16SrXXIII groups (Nejat et al., 2009a) (Table 1).

Some phytoplasma subgroups can infect multiple plant species and/or varieties of palm (Table 1). In Mexico, subgroup 16SrIV-D was found in *S. mexicana* and *Pseudophoenix sargentii* in the same area as 16SrIV-A in *C. nucifera* and *T. radiata* (Vázquez-Euán et al., 2011). Further, a palm species may host more than one subgroup of phytoplasma (Table 1) (Ntushelo et al., 2013c). In Mexico, samples from a single *S. mexicana* palm, a species initially thought to be resistant to LY (16SrIV-A) (McCoy et al., 1983) were found to contain both 16SrIV-A and 16SrIV-D subgroups (Vázquez-Euán et al., 2011). This diversity of phytoplasmas is also common in other phytoplasma groups and crops (Fránová et al., 2013).

### Pathogen detection

The need for reliable rapid detection methods for phytoplasmas is well documented (Lee et al., 2000; Bertaccini et al., 2014). Rapid detection in plants and vectors has resulted in many research advances, including screening of putative vectors and is now a vital part in the research of phytoplasma-associated diseases (Duduk and Bertaccini, 2011; Marcone, 2014). The relative ease of detecting phytoplasmas has lead one researcher to quip that “LY can arise spontaneously, anywhere in the world, whenever a phytopathologist appears with a PCR machine” (Harries, 2002). When sampling for phytoplasmas, Harrison et al. (1999) found that testing the immature leaves from around the apical meristem, which is rich in phloem, is the most reliable source of phytoplasma detection in palms. However, once palms are symptomatic, PCR testing of the phloem from the palm trunk (drilling a hole 10–15 cm into the trunk) is a non-destructive method of successful phytoplasma detection (Harrison et al., 2002b).

A internet based system named *iPhyClassifier* is available to assist in taxonomy which can analyse RFLP results using records from the GenBank (Zhao et al., 2009). The current sequence of single phytoplasma identification involves: DNA extraction; PCR amplification; nested PCR; sequencing; sequence analysis and assembly followed by identification using a barcode system (Makarova et al., 2012; Paltrinieri et al., 2015). This method will find the most abundant phytoplasma present, however when mixed infections are suspected, cloning before sequencing or deep amplification sequencing are required (Nicolaisen et al., 2011; Contaldo et al., 2012). The loop mediated isothermal amplification (LAMP) technique is becoming increasingly important as a rapid diagnostic tool for phytoplasmas, which is capable of being used to process large numbers of samples cheaply and is reportedly suitable for field use (Fukuta et al., 2003; Tomlinson et al., 2010; Hodgetts et al., 2011; Yankey et al., 2011; Keremane et al., 2015; Kogovšek et al., 2015). One main advantage over conventional PCR (and the often nested-PCR protocols) is that the LAMP protocol can be completed in less than 1 h on a simple (heatblock) or using more sophisticated equipment (GenieII, Optigene and quantitative PCR platforms) giving a digital display of data.

### Spread of lethal yellowing diseases

Vector transmission is considered the main form of spread of LYD. Phytoplasmas are known to be spread through the movement of plant material and phytoplasmas have been detected in the embryos of palm trees; although seed transmission has not been demonstrated (Cordova et al., 2003). In a new outbreak, LYD symptoms initially appear in one or two palms (foci or primary spread) followed by random cases up to 100 m away from the initial infection (secondary spread). This can be followed by a “jump” anywhere from a few kilometers to a 100 km (McCoy, 1976; Arellano and Oropeza, 1995; Nkansah-Poku et al., 2009; Bonnot et al., 2010). The rate of spread is often irregular with peaks occurring in some months but not consistently in all areas. Attempts to model spread are difficult on a small scale (Bonnot et al., 2010), but have been carried out at larger scales (McCoy, 1976). The rate of spread can be affected by geographical features such as mountain ranges that vectors may not be able to naturally cross (Arellano and Oropeza, 1995; CABI, 2012). Human activity has been an important cause of the spread of LYD. For example, Dollet et al. (2009) refer to grasses and palm trees imported from Florida for new golf courses in Mexico that may have harbored vector species in the 1980s when LYD first appeared in Mexico before spreading to Central America. Other crops have a similar history of human-aided spread of phytoplasma diseases (Bertin et al., 2007). Human activity is playing an increasingly important role in the spread of plant diseases globally (Burdon et al., 2006).

### Alternate plant hosts of lethal yellowing diseases

Many phytoplasmas have multiple plant host species, some of which are non-symptomatic (Marcone, 2014). Alternate plant hosts include both crop and non-crop species from which the vector(s) may acquire the phytoplasma. Identifying alternate hosts is difficult for non-symptomatic plants as detection by PCR does not determine if the phytoplasma is present due to a viliferous insect having fed on the plant or if the plant is an alternative host of the phytoplasma (Lee et al., 1998a).

In Florida many different palm species have been identified as hosts of LY (16SrIV-A) (Table 2). *Pandanus* species have also been identified as hosts. Few non-palm related species have been positively identified as alternate hosts to LYD associated phytoplasmas. In Mozambique a novel group of LYD associated phytoplasma was found in a mixed infection with a subgroup 16SrXXII-A in a palm plantation that was closely planted to pine trees. The novel group was closely related to “*Ca*. P. pini” (16SrXXI) which suggests that the phytoplasma was able to infect both the pine trees and coconut palms (Bila et al., 2015b).

In Jamaica, phytoplasma group (16SrIV-A) was positively detected in weeds *Emilia fosbergii* Nicolson and *Synedrella nodiflora* (L.) Gaertn. (Asteraceae) commonly found around plantations (Brown et al., 2008). Other weeds *Stachytarpheta jamaicensis* L. (Vervine), (Verbenaceae), *Cleome rutidosperma* DC (Cleomaceae) and *Macroptilium lathyroides* (L.) Urb. (Fabaceae) were also positively sampled for the 16SrIV-E subgroup although other subgroups were also identified (Brown and McLaughlin, 2011). In Ghana a large number of species were sampled but none were confirmed as an alternate host (Yankey et al., 2009) although Danyo (2011) listed *Desmodium adscendeus* DC. (Fabaceae) as having tested positive to Cape St. Paul Wilt Disease by PCR.

### Phytoplasma interactions with host plants and vectors

**Figure 2:**
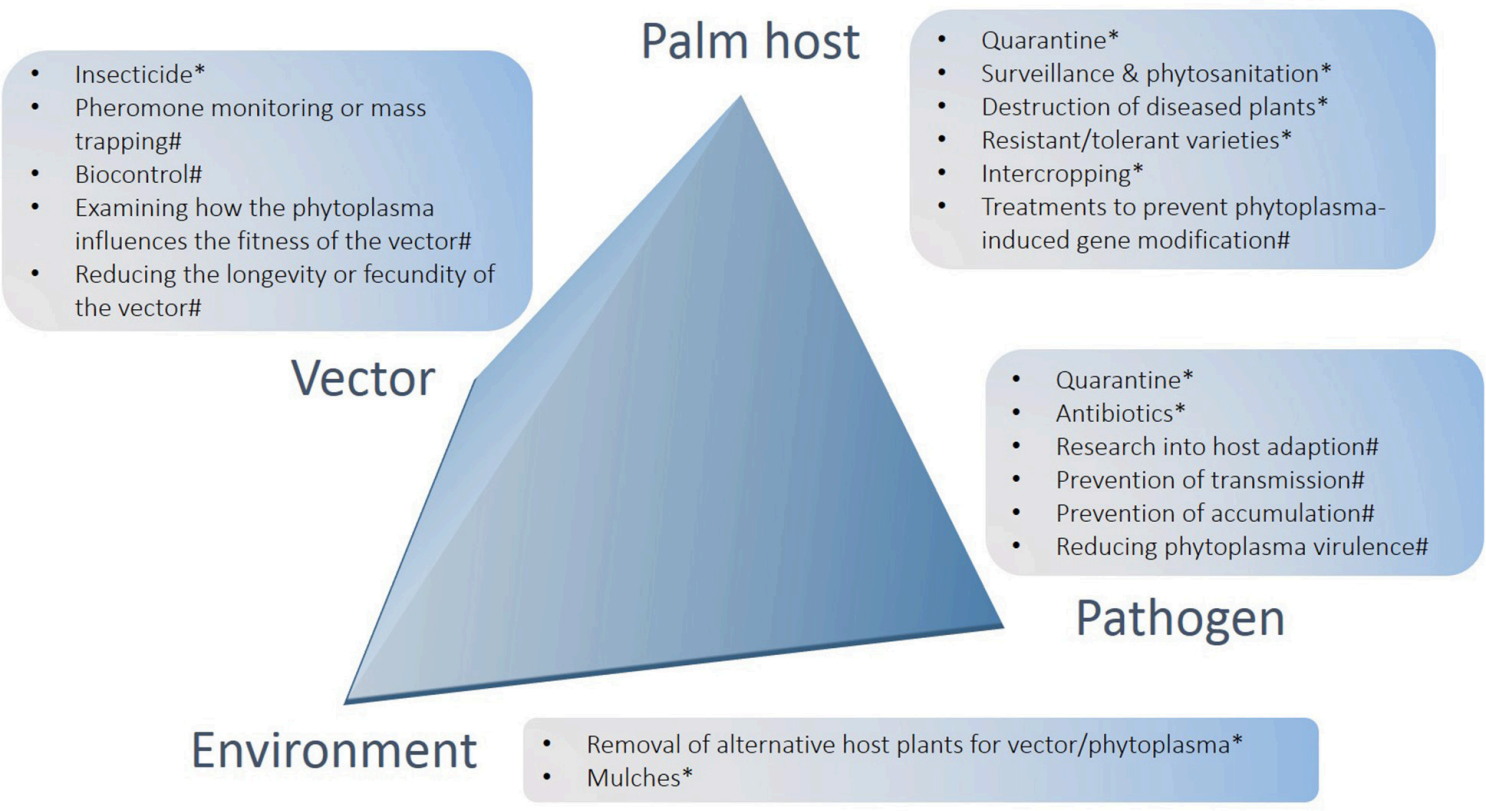
**Summary of options for the management of lethal yellowing diseases of palms arranged in relation to crop, pathogen, environment and vector components of the pathosystem**. ^*^Denotes options that have had impact in at least some field settings, including methods that have scope for further development and use; ^#^denotes potential future options.

Most vectors of phytoplasmas are from the Order Hemiptera, sub-order Auchenorrhyncha, except some species of the Family Psyllidae, which are in the sub-order Sternorrhyncha (Howard, 2001b) (Table 3). Many Auchenorrhyncha are known to feed on palms and, whilst they rarely cause direct damage to the palms, their ability to vector palm diseases makes them a significant threat (Wilson, 1987; Howard, 2001b). In some cases insect feeding can cause disease symptoms in palms, such as Finschhafen disorder (FD) in coconuts in PNG (Gitau et al., 2011). Examining the interactions between phytoplasmas and their insect and plant hosts opens possibilities of modifying these interactions for disease management (Figure 2) (Weintraub and Gross, 2013). The ability of phytoplasmas to live and multiply in both insect and plant hosts (Figure 2) involves adapting to different environments (Bai et al., 2006; Oshima et al., 2013). The latent period after transmission before symptoms appear or the phytoplasma is able to be re-transmitted, is suspected to be the time that the bacterium is shifting its metabolism to adapt to the new host and multiplying (Pacifico et al., 2015). One area of research that has been suggested involves examining how the phytoplasmas colonize their host so that strategies to prevent infection may be developed (Bertaccini et al., 2014). In some species, phytoplasma infections have been found to change the expression and signaling levels of certain host plant genes resulting in changes to floral development (Hoshi et al., 2009; Himeno et al., 2011). In studies of Aster yellows phytoplasma, it was found that 74 genes in the vector and 34 genes in the plant host were up regulated after infection, showing that genetics plays an important role in host adaption (Makarova et al., 2015).

**Table 3:** **Summary of knowledge on plant-phytoplasmas-vector interactions**.

**Location**	**Disease common name**	**16S rRNA Subgroup**	**Vector**	**Status**	**Testing method**	**References**
Florida	Lethal yellowing	16SrIV-A	*Haplaxius crudus* (formally *Myndus crudus*) [Cixiidae]	Confirmed	Cage transmission tests	Howard et al., 1983; Harrison et al., 2008
Mexico	Lethal yellowing	16SrIV-A	*H. crudus*	Suggested	Observations	Vázquez-Euán et al., 2011; Córdova et al., 2014
Jamaica	Coconut lethal yellowing	16SrIV	*Cedusa* spp. [Derbidae]	Putative	PCR and epidemic corresponding with pest outbreaks	Brown et al., 2006
Ghana	Cape St. Paul Wilt	16SrXXII	*Myndus adiopodoumensis* (Ceotto and Bourgoin, 2008) [Cixiidae]* Diostrombus* spp. [Derbidae]	Negative Putative	Cage trials One insect detected positive by PCR Cage transmission so far unsuccessful	Philippe et al., 2009 Pilet et al., 2009 Philippe et al., 2009
Mozambique	Coconut lethal yellow syndrome	16SrXXII	*Platacantha lutea* Westwood, 1837 [Pentatomidae]	Putative	PCR	Dollet et al., 2011
Tanzania	Coconut lethal disease	16SrIV-C	*Diastrombus mkurangai* Wilson [Derbidae] *Meenoplus* spp. [Meenoplidae]	Putative	PCR	Mpunami et al., 2000
India	Kerala wilt disease or Root wilt disease^*^	16SrIV-C Or IX	*Stephanitis typica* (Distant) [Tingidae] *Proutista moesta* (Westwood) [Derbidae]* Sophonia greeni* (Distant) [Nirvanidae]	Positive Positive Putative/ negative	Cage transmission Cage transmission Survey/PCR	Mathen et al., 1987, 1990 Rajan, 2013 Rajan, 2013
Sri Lanka	Weligama coconut leaf wilt disease	16XI	Multiple	Putative	Survey/PCR	Kumara et al., 2015
PNG	Bogia coconut syndrome		*Zophiuma pupillata* [Lophopidae] *Proustia* sp. (sic; >*Proutista* sp.) [Derbidae]	Putative	PCR of whole insect bodies	Pilotti et al., 2014

* *Some papers refer to Kerela wilt disease (KWD) and Root (wilt) disease (RWD), as being synonymous (Howard, 2001b; Sharrnlla et al., 2004), whilst PCR testing has classified KWD as 16SrIV-C (Edwin and Mohankumar, 2007a) and RWD as 16SrIX (Manimekalai et al., 2010). Sharrnlla et al. (2004) referred to “Kerala wilt disease of coconut palms, formerly named as root (wilt) disease” and considered it as a “separate group in the 16Sr classification” but did not indicate which group name. Another paper used PCR analysis and called KWD as closely related to the 16SrIV-C sub-group (Edwin and Mohankumar, 2007a). Manimekalai et al. (2010) reported that a new phytoplasma sub-group was found on diseased coconut palms and referred to the disease as RWD and stated “Our RWD phytoplasma sequence does not match an earlier reported Kerala (wilt) coconut phytoplasma sequence (AY158660) and the latter sequence does not have similarity with any known phytoplasma sequences in the database.” This phytoplasma was declared to be in the 16SrXI group which was also the cause of the sugarcane white leaf phytoplasma also commonly found in India (Manimekalai et al., 2010). This work was confirmed by Manimekalai et al. (2014a) and further sub-grouped into 16SrXI-B by Manimekalai et al. (2014c) who stated “The RWD phytoplasma sequence reported here did not show identity with sequence reported earlier for Kerala wilt coconut phytoplasma (GenBank Acc No AY158660) (from Sharrnlla et al., 2004), and which did not have similarity with any known phytoplasma sequences in the database.” Yadav et al. (2015) states they found 3 possible sub-groups interacting; 16SrXI-B in Kerala, XIV-A in Karnataka and another 16SrXIV in Kerala. Most literature on vectors refers to RWD only. The review paper Ramjegathesh et al. (2012) states that S. typica and P. moesta are both vectors of RWD which is consistent with the website for Central Plantation Crops Research Institute in India where most phytoplasma research is conducted (http://cpcri.gov.in/index.php?option=com_content&view=article&id=117&Itemid=142) however, Edwin and Mohankumar (2007a,b) suggest that S. typica is not a vector of KWD*.

In plants, phytoplasmas move through the phloem and are found primarily in the sieve elements of infected plants (Lee et al., 2000). Phytoplasmas are obligate parasites; they have a reduced genome and lack a cell wall. The structure of the phytoplasma genome has been the focus of much research (Christensen et al., 2005; Bai et al., 2006; Kube et al., 2012). The small repetitive phytoplasma genome lacks some metabolic functions as they obtain their nutrients from their hosts (Chen et al., 2012). In palms, LYD symptoms are thought to be a result of a disruption in the photosystem II reaction center efficiency and the carbohydrate transport system in the phloem (Maust et al., 2003). In India, studies found an increase in production of a superoxide anion and hydrogen peroxide in coconut affected by RWD prompting the suggestion that this knowledge could be used to manipulate the palms to somehow create a stronger resistance response (Sunukumar et al., 2014).

Management of the phytoplasma also depends on the relationship between the vector and the plant host. If the vector can complete its lifecycle on the plant host and the insect can reacquire the infection from that plant host it is more likely an outbreak will occur. In some phytoplasmas, infection and life cycle completion can occur only in certain non-crop species and the crop affected is a “dead end” host; that is, the vector cannot acquire the phytoplasma from that host. One example is *Bois noir* disease of grapevines in Europe (Foissac and Wilson, 2010). Vector species that feed on palm foliage do not always complete their lifecycle on palms (Wilson, 1987; Howard, 1995b, 2001b). In some publications palms are referred to as being susceptible to LYD only when mature. This, however needs to be interpreted in relation to the ecology of the vectors, which in some cases feed only on mature palms as adults and are rarely found on immature palms (Howard, 2001a). Thus, in cases where palms are referred to as being susceptible at all growth stages, for example BCS in PNG (Kelly et al., 2011), this could reflect the biology of the vector species.

Recent studies have found that transovarial phytoplasma transmission can occur. Phytoplasma infected vectors were reared and the offspring were hatched and reared on healthy plants. The offspring were found to be carrying the phytoplasma and able to transmit it to the healthy plants (Alma et al., 1997; Hanboonsong et al., 2002; Weintraub and Beanland, 2006).

Insect vectors, especially polyphagous species, have been shown capable of acquiring more than one phytoplasma (Danielli et al., 1996; Brown et al., 2006; Rashidi et al., 2014) but these are mostly closely related phytoplasmas (Lee et al., 1998a). In a study with the leafhopper *Euscelidius variegatus* (Kirschbaum) (Cicadellidae: Deltocephalinae), it was found that when a dual infection of the phytoplasma subgroup 16SrI-B or chrysanthemum yellows (CYP) and subgroup 16SrV or Flavescence dorée phytoplasma (FDP) occurred, CYP was not affected by FDP, while FDP was suppressed by CYP regardless of the order of infection. The mechanism of this suppression seemed to occur in the salivary glands where FDP was unable to multiply, CYP had a shorter latent period allowing it to overcome FDP (Rashidi et al., 2014). Further research into interactions between multiple phytoplasmas help explain variations in the pattern of spread of a phytoplasma in different regions when multiple phytoplasmas may be present (Rashidi et al., 2014).

Some phytoplasma infections have been shown to reduce the fitness of the vector, reducing longevity and fecundity (Madden and Nault, 1983; Bressan et al., 2005; D'Amelio et al., 2008; Malagnini et al., 2010) or body size (Mayer et al., 2011). Conversely, Aster yellows phytoplasmas (AYPs) have been shown to not only increase the fecundity of leafhoppers but also to increase their host range preference (Beanland et al., 2000; Kingdom and Hogenhout, 2007). In cases where phytoplasmas improved the longevity and fecundity of the vector it has been speculated that this reflects a longer association between the vector and phytoplasma such that the relationship had become mutualistic (Madden and Nault, 1983; Beanland et al., 2000; Ebbert and Nault, 2001).

In some pathosystems there is evidence that phytoplasma infected plants are more attractive to insect vectors than are healthy plants (MacLean et al., 2014; Krüger et al., 2015); however *Cacopsylla picta* (Foerster) (Hemiptera: Psyllidae) infected by *Ca*. P. mali preferred to oviposit on un-infected over infected apple trees (Mayer et al., 2011). Examination of the genome of Aster yellows phytoplasma “witches” broom (AY-WB) found that phytoplasma infection results in virulence “effectors” being secreted from the phloem which lead to changes in plant development that increases plant susceptibility (Sugio et al., 2011a,b; Hogenhout, 2013).

### Vector identification

Vector transmission is the most important route for phytoplasma dispersion (Rashidi et al., 2014) therefore has been the subject of much research aiming to deliver disease management (Duduk and Bertaccini, 2011; Bertin and Bosco, 2013). However, the vectors of many phytoplasmas have not been identified (Weintraub and Beanland, 2006; Krishnareddy, 2013). Methods for identifying vectors of phytoplasma are widely published (Howard, 1995a; Marzachi et al., 1998; Weintraub and Beanland, 2006; Weintraub and Wilson, 2010; Bertin and Bosco, 2013; Bosco and Tedeschi, 2013). A key issue is that because vectors need to have the ability not only to harbor the phytoplasma but also to re-transmit the phytoplasmas into another host plant, a positive PCR detection of phytoplasma in an insect does not mean that insect is a vector. For an insect to be a vector of a phytoplasma, the phytoplasma needs to be able to pass through the midgut and replicate within the insect. The phytoplasma then needs to be able to enter the salivary glands and accumulate to a high titer to be able to infect the host when the insect is feeding. Accordingly, vector research tends to follow a similar pattern of work beginning with identifying potential species in the range of the disease that are capable of transmitting a phytoplasma, i.e., phloem feeding species (Weintraub and Beanland, 2006). The insects are sampled from host and non-host plants, identified and first tested for the presence of phytoplasma using PCR with general phytoplasma primers or using primers selective for the particular phytoplasma that is being investigated (Eckstein et al., 2014). A positive test then makes that species a putative vector (Pilotti et al., 2014) but vector status cannot be established until transmission trials take place. It is commonly acknowledged that a successful transmission test method is logistically difficult. Much research has gone into finding better methods. For example, in New Zealand, field collected *Zeoliarus oppositus* were fed on *Ca*. P. australiense symptomatic *Coprosma robusta* “karamu” plants before being transferred onto disease free *C. robusta* and *Cordyline australis* (New Zealand cabbage tree). Although transmission was successful it was at a much lower rate than when using insects that were directly from the field. The reasons for this were suggested to be that the timing was inadequate to allow the insects become infective, or the sampling and feeding treatments affected the fitness of the insects (Winks et al., 2013).

Transmission tests of LYD vectors are especially difficult, involving caging mature palms during the incubation period and capturing thousands of insects that have been previously exposed to phytoplasma infected palms (Howard et al., 1983; Rajan, 2013). Though conventional vector transmission trials for LYD in palms have been carried out since the 1960s, very few have been successful (Tsai, 1980; Eden-Green, 1995), so knowledge of vectors is very incomplete and tentative (Table 3). To date, the only positively identified vectors of LYD are the planthoppers *Haplaxius crudus* (van Duzee), previously *Myndus crudus*, in Florida (Howard et al., 1983) and *Proutista moesta* (Westwood) in India (Rajan, 2013). There have been a great number of unsuccessful palm phytoplasma vector transmission trials (Tsai, 1980; Danyo, 2011). The seeming lack of information about vectors of LYD is also due to the cost involved in transmission testing and the fact that LYD are most commonly found in countries with few resources for research (Baudouin et al., 2009). Reflecting the logistical challenges involved in transmission tests, another form of vector testing that has been more recently developed involves using a sucrose based feeding medium. The vector is held in a small vessel and is able to access the feeding medium by piercing a Parafilm M® barrier to access a second compartment. Later, the medium can be tested for traces of phytoplasma by PCR. This shows that the insect is producing the phytoplasma in saliva and able to transmit it when feeding so is strongly suggestive of a capacity to transmit the pathogen when feeding on a plant host (Tanne et al., 2001).

## Disease management

### Surveillance and destruction of infected palms

Currently there is no cost-effective, curative treatment for LYD; however there have been some successes in keeping outbreaks at manageable levels. Black's approach, an integrated pest management and disease (IPDM) method, pioneered by a palm grower, Michael Black in Jamaica has been the most successful in reducing the incidence of LYD (Serju, 2012; CARDI, 2013). Black's approach includes on-farm quarantine, strict weekly surveillance, cutting down and burning of palms with LYD symptoms and replanting with a variety selected for high yield and LYD resistance as well as whole-farm weed control and a good fertilization regime (Myrie et al., 2011; CARDI, 2013). A multi-year comparison of seven farms showed a significant reduction in the number of palms affect by LYD on the four farms using Black's method, whilst three farms that had not employed any management continued to be devastated by the disease. Daily ground surveillance of parts of the plantation ensured that the entire plantation was covered every few weeks (Serju, 2012). At the end of the study, one farm lost only 10 trees out of 62,000 in 2010 whilst other farms lost thousands per year (Myrie et al., 2011; Serju, 2012).

In other parts of the world, the immediate removal of diseased palms is recommended. The slow spread of LYD in the Dominican Republic has been attributed to the early implementation of an eradication program combined with natural barriers preventing vector movement and an abundance of non-host palms (Martinez et al., 2008, 2010). In Ghana, aerial surveillance has been used to detect infected palms, which appear yellow against green canopy of healthy palms, followed by on ground inspection. Potentially the advent of relatively inexpensive unmanned aerial vehicles (UAV), “drones,” fitted with cameras will greatly assist in making plantation-scale surveys. Multi-spectral imaging, which can be drone-mounted or make use of other aircraft, also offers scope to support detection and mapping of disease (Hill et al., 2009) but does not yet appear to have been employed for LYDs. The immediate removal of infected trees, and replanting with a resistant variety has slowed the spread of the disease (Nkansah-Poku et al., 2009). Destruction of the felled palm is not usually mentioned in the literature, except in Jamaica where the felled palm is burned (Serju, 2012). Felled palms would rapidly lose turgidity and cease to be attractive to vector adults suggesting that destruction is not necessarily essential. Reflecting this, trials in Ghana showed that felling and insecticide treatment was not significantly more effective than felling alone in preventing spread (Nkansah et al., 2005).

### Quarantine

LYD can spread amongst close growing palms and also by “jumps” of up to hundreds of kilometers. Natural landscape barriers can inhibit vector movement and though leafhoppers can fly, “jumps” are most likely a result of human activity as in Mexico where grasses imported for landscaping are thought to have carried the vector to that country (Harries et al., 2001; Dollet et al., 2009). Quarantine protocols to prevent the human aided movement of LYD to new areas need to include restrictions on the movement of non-palm host plants such as grasses rather than being confined to palm seedlings (Harries et al., 2001). Although this may seem obvious, and that very strict phytosanitary regulations apply in many parts of the world, casual observations at airports and sea ports in developing countries (GMG, *pers obs* January 2016) show that the movement of a plant material in passenger luggage is not uncommon, especially for domestic, including island-to-island, travelers. Although phytoplasma has been detected in coconut embryos by PCR, there is not yet any evidence that disease occurs in the progeny of diseased palms (Nipah et al., 2007). Whilst this means that phytoplasma spread via the germ line of plants is unlikely, movement of vegetative propagules that can contain phytoplasmas (for example, cuttings and young plants) presents the risk of infected plants being introduced to a new area where, if competent vectors are present, a new epidemic could eventuate.

### Antibiotic treatment

Antibiotics have been shown to prevent or control phytoplasma infection in individual host plants by injection of tetracycline-type products into the trunk. Control requires a bi-weekly systemic treatment on a 4-monthly schedule which is not practical or affordable for commercial production but has been used for ornamental or valuable palms such as in tourist sites or hotels (McCoy et al., 1976; Eziashi and Omamor, 2010). The use of antibiotics in agriculture is banned in some countries including most of Europe (Musetti et al., 2013). Due to the expense of these treatments, and perceived health risks, which are often highly publicized in the media, antibiotics have never been considered a sustainable form of management except for protecting highly valuable ornamental trees (Been, 1995).

### Vector management

#### Alternate vector hosts

Management of alternative hosts of a vector is an important form of managing phytoplasma disease, particularly if the vector is univoltine (Belien et al., 2013). The immature stages of Cixiidae including the known LYD vector *H. crudus*, develop in the root zone of herbaceous plants, whilst the adults live on palms and trees. It is only adult *H. crudus* that are associated with palms or other monocotyledonous plants (Howard, 1995b). Howard and Oropeza (1998) reported 37 species of Poaceae and 4 species of Cyperaceae that had been identified as nymphal hosts of *M. crudus*. These included grasses such as St. Augustine or buffalo grass (*Stenotaphrum secundatum* (Walt.) Kuntze), and Guinea or Panic grass (*Megathyrsus maximus* Jacquin (Jacq.) B. K. Simon and S. W. L. Jacobs). Grasses in plantations need to be replaced with non-host species that are also shade tolerant. Legumes and other ground covers have been investigated to be used alongside other management including use of palm varieties resistant to the vectors (Howard, 1986, 2001b). Grasses that least favored *H. crudus* development included *Brachiaria brizantha* (Hochst. ex A. Rich.) Stapf., *Chloris gayana* Kunth and *Hemarthria altissima* (Poir.) Stapf and C. E. Hubb (Howard, 1995b).

When rearing *M. crudus* for research it was found that some types of mulches of coconut frond, fine pine or eucalyptus resulted in higher adult emergence by improving conditions in the soil for developing nymphs or were preferred by ovipositing females. Application of course materials such as bark nuggets, in contrast, resulted in less vector adult emergence (Howard and Oropeza, 1998). However, using mulches that inhibit the vector was considered prohibitively costly.

#### Insecticides

Vector control by spraying or trunk injection is used in some crops but has not been economically successful in coconut plantations (Been, 1995). In contrast to annual and temperate crops that might need protecting only for short periods of vulnerability, palms are long lived and vector feeding that results in transmission can be anytime during the palm's long productive life. A wide variety of insects feed on palms (Howard, 2001a; Gitau et al., 2009) so widespread use of insecticide could disrupt natural regulation of some of these by predators and parasitoids. Furthermore, insecticides need to be both fast acting enough to kill the vector before transmission can occur and yet enduring to protect the palms over longer periods (Riedle-Bauer et al., 2014). However, persistent insecticides are often damaging to human health and the wider environment (Eskenazi et al., 2007; Stehle and Schulz, 2015). Effective and acceptable-risk use of insecticides in palm plantations would require careful monitoring to be certain the vector is present, but this is precluded by the lack of knowledge of vector species of LYDs in many regions. A further issue is the paucity of technical support to smallholder farmers for effective monitoring (Weintraub, 2007; Bertin and Bosco, 2013; CARDI, 2013).

Notwithstanding the difficulties of insecticide use, “hot-fogging” with insecticides coupled with the felling of diseased palms reportedly slowed the spread of disease in some areas of Ghana (Nkansah et al., 2005). However, there was no difference in survival between the insecticide plus felling treatments and felling alone so the latter was considered to be the most economically and environmental sound method for small scale farmers (Nkansah et al., 2005). In India, attempts to prevent infection of newly planted seedlings in areas endemic with KWD through regular spraying or application of soil applied insecticides were unsuccessful despite the reduction in vector populations (Rajan, 2013). Similar results were found in Florida where a reduction in the vector population gave a significant reduction in the spread of the disease, but did not entirely prevent infection (Howard and McCoy, 1980; Been, 1995).

#### Vector trapping

Research in fruit orchards has exploited the volatiles responsible for the attraction of vectors to phytoplasma infected plants in traps for monitoring and mass trapping of vectors, and could be potentially be coupled with repellent compounds for use in “push-pull” strategies (Eben and Gross, 2013). The development of species-specific pheromone would also allow trapping but these approaches demand that vector species are known for a given pathosystem. Where possible, these species-specific approaches avoid the complications associated with the need for ongoing insect identification in surveillance programs that use sticky traps or other sampling methods (Eben and Gross, 2013). *H. crudus* adults can be monitored using sticky traps, especially those colored blue and white (Cherry and Howard, 1984), but many other arthropods, including other Auchenorrhyncha, are also attracted to such non-specific traps.

### Host plant resistance and replanting

Phytoplasma “resistance” has been described as the absence of symptoms associated with a low pathogen titer in the infected plants whilst phytoplasma “tolerance” is mild symptoms under a high pathogen titer (Jarausch et al., 2013). No genotype of coconut has yet been found to be totally “resistant” to LYD (Baudouin et al., 2009), though Harries (2002) describes “field resistance” as “the ability to plant…and survive long enough to repay the loan, cover costs and allow some profit.” A more formal description is the percentage of a population surviving after field exposure to the disease (Been, 1995).

Genetic improvement in coconut is difficult as it takes several years for a palm to be reproductive and relatively few seeds are produced per plant (Cardeña et al., 2003). Resistance screening of current genotypes of coconut is also difficult due to the long period between infection and the occurrence of symptoms, although, with PCR technology identification of infected palms is faster and more accurate. In some situations where resistance screening has taken place in coconut, it has been speculated that genetics were not necessarily the cause of the resistance as populations had succumbed to LYD when grown in a different locations (Mpunami et al., 2002). Although it is known that environmental conditions will affect the level of resistance (Baudouin et al., 2009), there is also the possibility that a resistant strain of coconut will encounter a different sub-group of phytoplasmas (or different vector species) when grown in a new location. This reinforces the need for more taxonomic identification (Mpunami et al., 2002; Baudouin et al., 2009; Odewale et al., 2012). Evaluation of the level of resistance also requires the deliberate transmission of the phytoplasma, which can be done by grafting in some crops (Carraro et al., 1998) but is not possible in coconut (Wallace, 2002) so requires studies using live vectors.

Testing resistance using vector transmission is closer to a real world situation, although the distinction between resistance to the vector or resistance to the phytoplasma cannot be easily distinguished (Jarausch et al., 2013); it requires monitoring of vector numbers in the lead-up to symptom development. In coconut, testing of resistance is usually performed by planting different varieties in a LYD endemic area (Baudouin et al., 2009; Odewale and Okoye, 2013). In PNG, the area in which BCS is expanding is home to the nation's coconut germplasm collection, forcing authorities to move representative germplasm to a safer (remote) site. This situation does present the likelihood of being able to identify any resistant or tolerant coconut varieties within the collection as the disease front reaches the site. A caveat of this approach, however, it does not allow resistance to be discriminated from “disease escape” (Mora-Aguillera, 2002). Disease escape occurs when susceptible plants remain uninfected, potentially the result of an anatomical feature such as thicker or hirsute cuticle. Whilst such traits may be useful in an IPDM program it is important to understand their relative strength in comparison with direct resistance to the pathogen because the durability of the overall resistance will be affected.

Genetic selection of coconut varieties has a long history (Baudouin et al., 2009). The most commonly recommended strategy for use is planting a range of resistant varieties in any growing area to reduce the risk of widespread plantation death should plant resistance be eroded by adaptation in the pathogen or vector population. Problems have occurred where varieties that have previously been reported as resistant have shown symptoms indicating a resistance breakdown (Broschat et al., 2002; Been and Myrie, 2005; Quaicoe et al., 2009). Variety improvement and evaluation needs to take place locally because resistance has been demonstrated to have a degree of site specificity; potentially due to the influence of environmental factors (Quaicoe et al., 2009; Odewale et al., 2012) and genetic differences between phytoplasma populations. The short generation times and small, repetitive genomes of phytoplasmas favor adaptation. To counter this, resistance gene stacking has been suggested to prevent rapid breakdown of resistant varieties (Bertaccini et al., 2014; Gurr et al., 2016). The very recent advent of clustered regularly interspaced short palindromic repeats (CRISPR)-based tools for gene editing are likely to allow rapid progress in understanding and exploiting the genetics of phytoplasma resistance traits because of the ease of gene insertion or silencing it allows (Belhaj et al., 2013). CRISPR-based methods also offer the advantages of being much cheaper and requiring less advanced training and infrastructure compared with former approaches for genetic manipulation (Baker, 2014). These factors make this new technology attractive in the context of LYD where much work needs to be done in developing counters and on a low budget. A further advantage is that because varieties produced by CRISPR-based manipulation need not contain transgenes, it appears that regulatory authorities in at least some jurisdictions will not consider such varieties to be genetically modified; an “anti-browning” mushroom developed using CRISPR, for example, is not subject to USDA approval (Waltz, 2016). This may have benefits for public acceptance. Finally, aside from the aforementioned challenges in genetic improvement of coconut, the production of seedlings on a large scale once resistance is identified is also difficult and slow, however improvements in tissue culture methods are showing promise that this process could be improved in the future (Nguyen et al., 2015).

A more general challenge associated with the use of host plant resistance to manage LYDs is that varieties with resistance to the phytoplasma may lack resistance to other serious biotic threats and this might geographically constrain their value. For example, two exotic ecotypes of coconut failed to survive in PNG due to attack by the rhinoceros beetles, *Oryctes rhinoceros* (Linnaeus), *Scapanes australis* (Boisduval) (Coleoptera: Scarabaeidae) and the black palm weevil *Rhyncophorus bilineatus* (Montr.) (Coleoptera: Curculionidae) (Ovasuru, 1994).

Notwithstanding the challenges, history does point to the potential of host plant resistance to contribute to LYD management. Cape Saint Paul Wilt Disease (CSPWD) in Ghana has been addressed by resistance screening work using a hybrid of Sri Lanka Green Dwarf (SGD) and Vanuatu Tall (VTT) which is being used for replanting following the disease epidemic (Quaicoe et al., 2009). Similarly, in Jamaica, after devastating losses to LYD, the local Jamaican tall variety was widely replaced with the Malayan dwarf varieties and MayPan, which led to a recovery of the coconut industry (Been, 1995; Harrison et al., 2002a). A new outbreak of LYD in Jamaica killed up to two-thirds of the Malayan and MayPan varieties, which triggered research exploring the possibility of the occurrence of a new phytoplasma, a new vector or a change in the virulence of the current phytoplasma. However, Broschat et al. (2002) cast doubt on the results of the original resistance trials, and showed new evidence that the MayPan and Malayan varieties were not resistant as previously claimed. It is now known that there are several subgroups of phytoplasma present in Jamaica (Ntushelo et al., 2013c).

Mass replanting as a prevention measure for LYD is considered to be economically and practically difficult because it is followed by a long period before trees mature sufficiently to yield nuts (Nkansah et al., 2005; Danyo, 2011). Reflecting this difficulty and independent of LYD, the average age of many palm plantations in many parts of the tropics is old, to the extent that yields are declining (Snaddon et al., 2013). Replacing older palms with higher yielding and disease resistant varieties would increase production but only in the medium to long term. Replanting sites must also be carefully designed so as not to interrupt ecosystem services such as erosion control and providing shade for other crops and amenity (Snaddon et al., 2013).

### Intercropping

To minimize the risk of LYD losses, particularly where only varieties with low or moderate resistance are available, intercropping coconut with other crop species can provide alternate sources of income and insurance against total crop failure and loss of income (Andoh-Mensah and Ofosu-Budu, 2012). Examples of potentially suitable species include cacao (*Theobroma cacao* L.) (Osei-Bonsu et al., 2002), *Citrus* spp., cereals, cassava (*Manihot esculenta* Crantz), sweetpotato [*Ipomoea batatas* (L.) Lam], peanuts (*Arachis hypogaea* L.), banana (*Musa* spp.), cashew nut (*Anacardium occidentale* L.), pineapples (*Ananas comosus* (L.) Merr.) or grasses for grazing (Godoy and Bennett, 1991; Andoh-Mensah and Ofosu-Budu, 2012). Though intercropping can result in reduced yield of coconut, total income is improved or only marginally affected (Godoy and Bennett, 1991; Andoh-Mensah and Ofosu-Budu, 2012). Forms of polyculture are traditionally practiced in many areas in which coconut is grown and serve to provide subsistence food products to smallholders. Polyculture is also possible in more intensive and commercial production systems and, though the economy of scale may be lower than in a monoculture, labor constraints can be eased by the spreading of need across several crops with contrasting requirements such as for harvest (Schroth and Ruf, 2014). An important caveat to the value of intercropping is that a given phytoplasma can attack multiple species so the choice of crops to be used needs to take this into consideration. For example, the phytoplasma associated with BCS has a high similarity with phytoplasma isolated from banana (Banana wilt associated phytoplasma, BWAP) and betel nut *Areca catechu* (L.) (Davis et al., 2012, 2015) and both of these crops are commonly grown with coconut palms. Betel nut is affected by another phytoplasma in India (16SrXI) that also causes LYD in coconuts (Manimekalai et al., 2010; Ramaswamy et al., 2013).

### Abiotic factors and climate change

Relatively little information is available how abiotic factors affect LYD (Hunt, 2002), what is know could be used to inform decisions on management aimed at reducing the spread or severity of LYD. It is known that the severity of phytoplasma associated diseases is a result of the interaction between temperature and moisture conditions in the host (Krishnareddy, 2013). Indian studies found that palms with root (wilt) disease had impaired stomatal regulation and this was associated with excessive water loss and leaflet flaccidity (Rajagopal et al., 1986). Aside from such factors that cause stress to the palms, making symptoms more pronounced, there seems to be no evidence that plant nutrition, spacing or other local management practices are able to reduce infection progression the severity of LYD. Vectors are affected by abiotic conditions including mulches (see above) and the density of host plants (including non-coconut palms and host grasses) and the distance between coconut plantations affects the rate of spread of LYD via vector dynamics. Climate and landscape have a major influence on the spread of LYD (Arellano and Oropeza, 1995; Mora-Aguillera, 2002). However, since there is little evidence that abiotic factors influence the incidence LYD, it is likely that the climate and landscape are influencing population and distribution of the vectors. In Mexico, the generally dry climate and the distance between plantations resulted in LYD outbreaks being less explosive than in Florida or Jamaica with their higher rainfall and higher density of palm plantations (Mora-Aguillera, 2002). Prevailing winds and geographical features such as mountain ranges also have a role in the spread of phytoplasma diseases and the direction of spread (Arellano and Oropeza, 1995; Mpunami et al., 2000; Mora-Aguillera, 2002).

Research with other phytoplasmas found that vector infectivity to plants is temperature and CO_2_ dependent. In chrysanthemum yellows and “flavescence dorée,” phytoplasma multiplication in insects was faster under cooler conditions (18–22°C; CO_2_ 400 ppm) but the opposite was found in plants where warmer conditions showed a faster phytoplasma multiplication (22–26°C; CO_2_ 800 ppm) (Galetto et al., 2011). An epidemiological study in Mexico examined foci points, dispersal gradients and sampling zones and suggested the possibility of LYD eradication from an area if foci points are determined early (Mora-Aguillera, 2002). Rapid detection technology plays an important role in early detection of foci points (Mora-Aguillera, 2002; Myrie et al., 2011; Yankey et al., 2011) so the use of remote sensing (see above) is likely to be more important in future LYD management.

Where several vector species are able to transmit a phytoplasma there is variation in the ability of each species to successfully transmit the phytoplasma (Bosco et al., 2007). Although phytoplasma titer in the vector is an indication of infectivity, Goodwin et al. (1999) suggested that disease incidence is better estimated by vector population levels. There is also a possibility that within a population there may be different transmission capabilities and that population manipulation may be a possible management strategy (Bosco et al., 2007).

Climate change is considered to have a significant impact on the spread and establishment of vectors and phytoplasma-associated diseases into areas with a previously unfavorable climate (Foissac and Wilson, 2010; Krishnareddy, 2013). Thuiller (2007) stated that a 1°C increase in temperature could shift ecological zones by up to 160 km. Increased temperatures have already been shown to result in insect species moving into new areas (Walther et al., 2002; Parmesan and Yohe, 2003) and even new countries. It is also known that elevated temperatures increase the rate of spread of some phytoplasmas either through faster multiplication in the host or higher feeding frequency of the insect vectors resulting in increased transmission opportunities (Maggi et al., 2014). As the temperature range of LYDs is not known it is difficult to accurately predict the consequences of climate change. It was speculated that since the vectors for LYD in Florida could be found further north (N29°) than the disease (N27°) and that temperature might limit the pathogen rather than the vector though the vectors were present at lower densities in the northern part of their range (Halbert et al., 2014).

Aside from warming, climate change is closely related to an increasing frequency of drought and storms that can increase stress on plants (Dale et al., 2000). There have been reports of a higher incidence of LYD after hurricanes in the Caribbean, due to increased stress on the palms. Even relatively resistant varieties of coconut have been found to succumb to LYD when plants are stressed (Hunt, 2002). Climate change and the resulting climate disturbances could result in an increase in the severity of many phytoplasma associated diseases including LYD.

## Conclusions and outlook

Phytoplasmas affect a large number of economically important crops worldwide, and this has spawned an extensive research literature (Bertaccini et al., 2014) in which it is clear that phytoplasmas have proven challenging to manage. This is evident in the foregoing accounts of LYD management in various continents in which attempts to understand fundamental aspect of the pathosystem such as the identity of the pathogen and its vectors often remain unresolved; an observation that applies generally to phytoplasmas (Bertaccini, 2015). Associated with this, effective management of LYDs has been achieved in only a limited number of locations and even these seem threatened by the possibility of genetic adaptation by the phytoplasma or arrival of a new strain. The wider phytoplasmology literature offers some cause for optimism in prospects for control. In France, for example, winegrowers have successfully used a coordinated approach to controlling leafhopper vectors of flavescence dorée (Verpy et al., 2013). In Iran, Witches' broom disease of lime, associated with *Ca*. P. aurantifolia, management has been supported effectively by a combination of the development of rapid detection technology and biological information of the interactions between the pathogen and host plant (Mardi et al., 2011). These cases illustrate the principle that biological information is key to effective management. Whilst phytoplasmas are more difficult to study than many other types of plant pathogen because they cannot be readily cultured *in vitro*, advances in PCR-based and LAMP detection are allowing rapid progress that will facilitate easier and cheaper tracking of phytoplasmas in plants and insects especially in the field. CRISPR-based approaches will support research into the genetic basis for host-pathogen-vector interactions and speed breeding efforts, whilst remote sensing using drones, potentially equipped with multi-spectral imaging systems, will facilitate rapid detection of symptomatic plants so help in outbreak management.

Some emerging fields also may be important in phytoplasmology. Endophytic microorganisms and their interactions with plants is a field that is poorly explored (Bianco et al., 2013) but of promise in phytoplasmology because of reports of spontaneous remission or recovery from phytoplasma infection. Fungal endophyte strains have been isolated from these plants, grown and reapplied to other infected plants in which they led to a reduction in symptom severity and a lower phytoplasma (*Ca*. P. mali) titer in plant host tissues (Musetti et al., 2012). Furthermore, various bacteria species are present as asymptomatic endophytes in plants such as grapevines and it has been hypothesized that these could be used as biocontrol agents for plant pathogens (West et al., 2010). Beneficial rhizospheric microorganisms also are being investigated; arbuscular mycorrhizal fungi (AMF) commonly form mutualistic associations and boost their host plant's ability to overcome abiotic and biotic stress. Trials found that a mixed inoculum of AMF improved plant growth and root development in daisy (*Chrysanthemum carinatum* Schousb.) when challenged with *Ca*. P. asteris (16Sr-1B) (Marzachi et al., 2012). In other work, a combination of the bacterium *Pseudomonas putida* and mycorrhizal fungus *Glomus mosseae* were used against a chrysanthemum yellows (CY) a phytoplasma infection of chrysanthemum resulting in a reduction in symptoms (Bianco et al., 2013). More widely, antimicrobial peptides are currently being trialed against other microbial diseases of grapevine and may find application in phytoplasma control (Rosenfield et al., 2010; Romanazzi et al., 2012; Spinas et al., 2012).

Advances in knowledge mean that the prospects for the development of novel control technologies of LYDs look good. Currently management of LYDs is based on detection of infections and immediate destruction of infected plants along with replanting using alternative crop species or any available palm varieties with resistance (Baudouin et al., 2009; Myrie et al., 2011). A major limitation of this strategy is that it relies on the appearance of visible symptoms, which, in coconut is often months after infection, a period during which vectors may have spread the pathogen widely. This highlights the need for research and development to apply newly-available technologies, particularly LAMP field kits and remote sensing, to support current management efforts, whilst success in plant breeding to provide longer term control is likely to be well supported by CRISPR-based tools.

## Author contributions

All authors listed, have made substantial, direct and intellectual contribution to the work, and approved it for publication.

### Conflict of interest statement

The authors declare that the research was conducted in the absence of any commercial or financial relationships that could be construed as a potential conflict of interest.
